# From design to 3D printing: A proof-of-concept study for multiple unit particle systems (MUPS) printed by dual extrusion fused filament fabrication

**DOI:** 10.1016/j.ijpx.2024.100299

**Published:** 2024-10-26

**Authors:** Lee Roy Oldfield, Aaron Felix Christofer Mentrup, Stefan Klinken-Uth, Tobias Auel, Anne Seidlitz

**Affiliations:** a Heinrich Heine University Düsseldorf, Faculty of Mathematics and Natural Sciences, Institute of Pharmaceutics and Biopharmaceutics, Universitätsstraße 1, 40225 Düsseldorf, Germany; b INVITE GmbH, Otto-Bayer-Straße 32, 51061 Cologne, Germany

**Keywords:** 3D printing, Fused filament fabrication, Dual extrusion printing, Multiple unit particle systems, Hot-melt extrusion, Additive manufacturing, Personalised medicines

## Abstract

MUPS (multiple unit particle systems) are oral dosage forms consisting of small particles which are filled into capsules or compressed into tablets. Compared to monolithic sustained-release tablets, MUPS tablets rapidly disintegrate inside the stomach releasing the contained small particles, which can be emptied from the stomach independent of housekeeping waves. Control of release can be achieved by adapting the particle composition. Despite the advantages of MUPS, only a limited number of preparations are available on the market. 3D printing could be a new advantageous method to produce MUPS tablets compared to the conventional production via tableting. Due to the increasing research interest in personalised medicine, especially regarding dose adjustments, this flexible production approach could be a promising concept. Therefore, this work proposes a concept for printing MUPS tablets using a dual extrusion fused filament fabrication 3D printer. The general idea is that the two print heads can be used independently to print a water-soluble tablet shell with the first print head and incorporate functional particles into the tablet shell with a second print head using different materials for each step. In this study, a modular four-particle-layered tablet computer model containing 196 cylindrical particles with a diameter of 1.4 mm, a height of 1.0 mm and a total tablet size of 22.6 × 8.5 × 6.0 mm is proposed. A first proof-of-concept study with drug-free commercially available polylactic acid filament for the particles and polyvinyl alcohol filament for the tablet shell revealed critical parameters (such as filament retraction, z-offset and water content of filaments) for the successful printing of the proposed computer model. In addition, the successfully printed model 3D-MUPS tablets and incorporated particles were characterised, revealing a reproducible manufacturing process. The printed model particles had a diameter of 1.27 ± 0.04 mm and a height of 1.05 ± 0.01 mm. One of the challenges of the new approach was to avoid particle agglomeration because of remelting processes during the printing with two print heads. 57.54 ± 18.59 % of the 196 printed particles were present as single particles. Finally, the transferability and suitability with a model API-loaded (paracetamol) hydroxypropyl methylcellulose filament for the particles and a polyvinyl alcohol tablet shell was successfully tested. On average, 80 % of paracetamol was released within 3 h (2–4 h). Overall, this work shows an innovative new manufacturing method for dose-adjustable personalised MUPS tablets but also considers new challenges arising from the different manufacturing processes.

## Highlights

• MUPS tablets are oral dosage forms consisting of small multiparticulates (< 2 mm).
• 3D printing can be a new manufacturing method for personalised MUPS tablets.
• A modular model with 196 cylindrical particles with a diameter of 1.4 mm is proposed.
• Dual extrusion 3D printing can be used to print 3D-MUPS with commercial filaments.
• The proof-of-concept study is transferable to API-loaded personalisable 3D-MUPS.

## Graphical abstract

**Figure:**
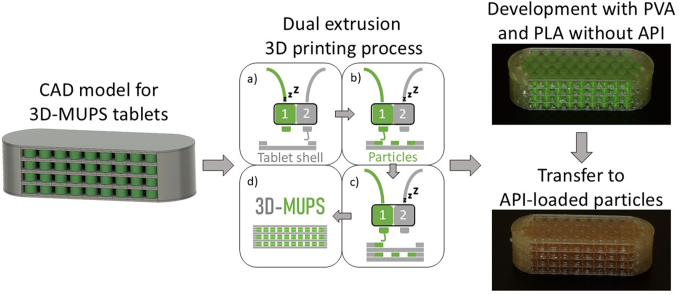

## 1 Introduction

Multiple unit particle systems (MUPS) are solid oral dosage forms in the form of tablets and hard capsules. MUPS consist of small multiparticulate units, such as pellets, either pressed into tablets with excipients or filled into hard capsules, forming a single dosage unit (Abdul et al., 2010). Pellets in MUPS preparations usually have a modified release and can be divided into coated or uncoated pellets. In the case of coated pellets, often drug-free starter cores are coated with an active pharmaceutical ingredient (API) and/or a functional coating. Uncoated pellets are typically matrix pellets in which the API is incorporated (Kallakunta et al., 2018).

MUPS are administered orally and disintegrate within a short time into their multiparticulate units (Abdul et al., 2010). There is contradictory evidence in the literature that particles smaller than 2 mm, commonly incorporated in MUPS formulations, have a faster gastric passage. It is assumed that small particles can leave the stomach through the pylorus, even in the fed state. In comparison, sustained-release monolithic tablets remain in the stomach and are only transported via the migrating motor complex in phases of highest motility (housekeeping waves) in fasted state (Newton, 2010). Another advantage of MUPS is the even distribution of small individual particles inside the gastrointestinal tract. Furthermore, products containing otherwise incompatible active ingredients or specific release kinetics can be realised by combining different pellet types in one tablet. In addition, multiple individual modified release units reduce the risk of dose dumping (Abdul et al., 2010; Bechgaard and Nielsen, 1978; Dukić-Ott et al., 2009; Kallakunta et al., 2018).

Despite all these advantages, only a limited number of MUPS preparations are on the market (Abdul et al., 2010; Kallakunta et al., 2018; Patel et al., 2017). Capsules filled with pellets are more expensive to manufacture than MUPS tablet preparations due to the reduced production throughput (Abdul et al., 2010; Clelik and Maganti, 1994). However, the production of MUPS tablets is complex and time-consuming. During the compression step, it is essential that the applied force does not damage the pellets. Cracks in functional coatings, for example, could have a negative impact on the desired release kinetics. In addition, the individual pellets may fuse irreversibly during the compression process and can no longer act as small individual particles (Abdul et al., 2010; Clelik and Maganti, 1994). To use the advantages as mentioned above, the search for alternative production methods has to be considered.

3D printing is becoming integral to pharmaceutical research for the production of various dosage forms. Especially in the field of personalised medicine, 3D printing has many potential advantages (Trenfield et al., 2018). The fused filament fabrication (FFF) method is particularly noteworthy in the case of 3D printed personalised oral dosage forms. It is a process in which a polymer in the form of a filament is melted and extruded through a nozzle before being deposited layer-wise onto a heated print bed (Jamróz et al., 2018; Zema et al., 2017). The process is very flexible, and by using computer-aided design (CAD) software, the object can be individually adapted, for example, to control the dose or release kinetics (Goyanes et al., 2014; Goyanes et al., 2017). The printing process is preceded by a hot-melt extrusion to produce filaments with the desired pharmaceutical polymers, APIs, other excipients and/or to control release kinetics (Tan et al., 2018).

Within the field of FFF printing, dual extrusion printing is a promising manufacturing technique for medicinal products with a complex design that includes the use of two filaments with different compositions. In this case, the printers are equipped with two print heads (Jamróz et al., 2018). This allows, for example, the use of polymers differing in release kinetics or incorporating different APIs while printing one object. This technology has already been used to print pharmaceutical oral dosage forms. Goyanes et al. have produced capsule-shaped multilayer and shell-core tablets (Goyanes et al., 2015). Okwuosa et al. printed core-shell tablets with the addition of a gastroretentive shell (Okwuosa et al., 2017). Kempin et al. also used this technique to print gastroresistant core-shell tablets, but with the addition of a thermolabile API at low printing temperatures (Kempin et al., 2018). Zhang et al. used this technique to print bilayer tablets (Zhang et al., 2022). Apart from oral dosage forms, individualised drug-eluting implants have also been proposed by Domsta et al. (Domsta et al., 2023), showing the versatility of this method. Dual extrusion FFF printing might, therefore, also be used to produce MUPS tablets.

One of the main challenges in printing MUPS tablets is the production of small particles with a diameter of less than 2 mm. In the past, attempts have been made to produce small particles using 3D printing. Krause et al. printed mini tablets for paediatric patients with a 1.5 to 4 mm diameter using FFF (Krause et al., 2021). Also, semi-solid extrusion has already been used by Hu et al. to print cylindrical API-loaded minitablets with a diameter and height of 3 mm for paediatric use (Hu et al., 2023). Awad et al. printed individual pellets with a 1- or 2-mm diameter using selective laser sintering (SLS) (Awad et al., 2019). Xu et al. used stereolithography (SLA) technology to print pellets in the size of 1- or 2-mm diameter (Xu et al., 2021). Most of the above-mentioned studies successfully printed particles below 2 mm. However, no studies are available so far, that show a one-step 3D printing process to manufacture MUPS. The printed particles by Awad et al. and Xu et al., for example, would need to be filled into a capsule in a subsequent step to form a MUPS. Even though SLS and SLA may offer a higher printing precision compared to FFF, the big advantage of FFF is its flexibility. As already mentioned, it is possible to print several polymers with different APIs by using FFF with multiple print heads without pausing the process. The incorporation of multiple APIs is more difficult to realise with SLS and SLA, as an intervention during the printing process may be necessary to switch/add powder materials or resin tanks (Awad et al., 2019; Robles-Martinez et al., 2019). Dual extrusion FFF could be advantageous here and add new manufacturing possibilities.

The aim of this study was to investigate the feasibility of producing multiparticulate systems with small particles of less than 2 mm in diameter using FFF dual extrusion printing. For this purpose, a modular computer design of the 3D-MUPS was created. The requirements for this model were that the highest possible particle loading should be achieved to enable future dose adjustment. A maximum tablet size of 23 × 11 × 9 mm was also defined based on marketed monolithic tablets (*ASACOL® Enteric Coated Tablets: Consumer Medicine Information*, 2023). In addition, the tablet should not have sharp edges to improve swallowability. The printed 3D-MUPS should have a rapidly disintegrating tablet shell and contain as many particles <2 mm as possible. The process was developed and tested with commercially available polyvinyl alcohol (PVA) and polylactic acid (PLA) filaments as part of a proof-of-concept study. Finally, the suitability and transferability of the approach were tested by printing a drug-containing MUPS. For this purpose, an API-loaded (paracetamol) hydroxypropyl methylcellulose (HPMC) filament was manufactured by hot-melt extrusion and used to print the particles.

To the best of the authors' knowledge, this is the first report of the engineering of a complete multiple unit particle system tablet via FFF.

## 2 Material and methods

### 2.1 Materials

Commercially available technical grade filaments with a diameter of 2.85 mm were used to establish the proof-of-concept experiments. Water-soluble PVA (Aquasolve PVA Formfutura®, Netherlands) was used for the printing of the tablet shell, and water-insoluble PLA (EasyFil PLA Formfutura®, Netherlands) was used for the drug-free particles. All filaments were stored under defined humidity conditions (relative humidity <25 %) inside a customised filament box filled with silica gel as a desiccant. The used filament box was built according to (*DIY Filament Box selber bauen – die ANYBOX V1, Ingenieurbüro Dr. Janko GmbH*, 2020). The relative humidity inside the box was tracked using a digital hygrometer. The filament was stored on a rotating rod in the box and was fed from the box into the gear of the 3D printer via another Bowden tube to achieve a closed system.

AFFINISOL™ HPMC HME 15LV (hydroxypropyl methylcellulose, HPMC) was kindly donated by Dow Wolff Cellulosics GmbH (Bomlitz, Germany) and was used as a polymer for hot-melt extrusion of the API-loaded filament. Aerosil® 200 (fumed silica) was used as a flowability enhancer for the powder feeding process and was purchased from Evonik Operations GmbH (Rheinfelden, Germany). The model drug paracetamol (PCM), also known as acetaminophen, was purchased from Atabay Kimya Sanayi Ticaret A.S. (Istanbul, Türkiye). Phosphate buffer pH 6.8, according to European Pharmacopoeia 5.17.1 (Ph. Eur., version: 11.1), was used for dissolution studies.

### 2.2 Methods

#### 2.2.1 Computer model design of the MUPS tablet

A CAD software (Autodesk® Fusion 360™, version: 2.0.17710, Autodesk, America) was used to develop the modular MUPS models. Two different models were generated, one for the tablet shell and one for the incorporated particles. The STL files were imported into the slicing software (UltiMaker Cura 5.1.0, Ultimaker B.V., Netherlands). The models for the preliminary first MUPS tablets, as shown in Fig. 2, were designed with a different CAD software (FreeCAD©, version: 0.19, America).

#### 2.2.2 3D printing of model MUPS tablets

A dual extrusion FFF 3D printer (UltiMaker S3, Ultimaker B.V., Netherlands) was used for printing of MUPS tablets. The used printer was a Bowden extruder with two print heads. One print head was equipped with a 0.25 mm diameter nozzle, while the other had a 0.4 mm diameter nozzle. The print heads move in the x and y-axis, and the print bed moves on the z-axis. Unless otherwise specified, each 3D-MUPS print was started with the active levelling of the print bed. The slicing software was used to adjust the printing parameters. Commercially available water-soluble glue was used to improve the print bed adhesion of the first layers. Ten batches of tablets were printed. Each batch contained three tablets.

Table 1 lists the most critical empirically determined printing settings for the slicing software. Print head 1 was used to print the particles, while print head 2 was used to print the tablet shell. The manufacturing process for 3D-MUPS was conducted as described in Fig. 3. For the proof-of-concept studies, the hot-melt extrusion step was omitted, and commercially available PLA and PVA filaments were used.

**Table 1:** Empirically determined printing settings in the slicing software for the dual extrusion printing process of 3D-MUPS, PLA: polylactic acid, PVA: polyvinyl alcohol.

Printing parameter	Print head 1 (particles)	Print head 2 (tablet shell)
Used polymer	PLA	PVA
Print head	UltiMaker AA 0.25 mm	UltiMaker BB 0.4 mm
Nozzle diameter [mm]	0.25	0.4
Printing temperature [°C]	190	224
Print bed temperature [°C]	60	60
Layer height [mm]	0.1	0.1
Printing speed [mm/s]	10	10
Support structure	skirt	skirt
Active bed levelling	activated	activated
**Additional settings print head 1:** retraction at layer change, maximum retraction count per layer: 300, retraction minimum travel: 0 mm		
**Additional settings print head 2:** retraction at layer change, Z seam alignment: random, infill pattern: Zigzag, Infill density: 100 %, walls printed before infill		

The examination of printed tablets showed undesired particle agglomeration due to thin polymer strands between the particles, which may have been caused by a remelting of the PLA particle's surface when the PVA printhead travelled across them in the next layer. In order to determine whether this was the case, three batches of a reduced CAD model were printed. These tablets consisted only of the lower PVA layers and one layer of particles. One PVA cover layer was printed above this reduced model in an alternating position.

The storage conditions for the PVA filament influenced the quality of the printed tablets. Undesirable printing results were observed when a higher relative humidity (r.H.) was measured. To test the effects of ambient humidity on the PVA filament, the filament was stored either in a filament box with a r.H. of <25 % or in a hygrostat chamber with a relative humidity of 57.5 %. The filaments were subsequently used for printing. Three tablets were printed with every filament under the same printing conditions. In addition, the first four layers of the tablet shell were printed three times to analyse the influence on the printing accuracy of the first layers depending on the different storage conditions. These tablets and PVA layers were analysed using a digital microscope (VHX-7000, Keyence, Japan). Images of the PVA layers were taken using a glass plate, illuminating the object with light from underneath and above to make the layers translucent.

#### 2.2.3 Characterisation of model MUPS tablets

##### 2.2.3.1 Optical appearance and dimensions of model MUPS tablets

Three printed tablets were visually analysed using a digital microscope (VHX-7000, Keyence, Japan). The dimensions of twenty tablets were determined using a digital calliper.

##### 2.2.3.2 Uniformity of mass of single-dose preparations according to Ph. Eur. 2.9.5

Twenty printed tablets were weighed and tested according to monograph 2.9.5: “Uniformity of mass of single-dose preparations” of the European Pharmacopoeia (version: 11.1). According to the monograph, for tablets (uncoated and film-coated) weighing 250 mg or more, no more than two units of 20 tablets may deviate by more than 5 %, and no unit may deviate by 10 % from the average mass.

##### 2.2.3.3 Friability of model MUPS tablets according to Ph. Eur. 2.9.7

Ten printed tablets were tested according to monograph 2.9.7: “Friability of uncoated tablets” of the Ph. Eur. (version: 11.1) with a friability tester (ERWEKA TA 120, ERWEKA GmbH, Germany).

##### 2.2.3.4 Resistance to crushing of model MUPS tablets according to Ph. Eur. 2.9.8

Ten printed tablets were tested according to monograph 2.9.8: “Resistance to crushing of tablets” of the Ph. Eur. (version: 11.1) with a tablet hardness tester (ERWEKA TBH 210, ERWEKA GmbH, Germany). The tablets were orientated with the short side towards the jaws.

#### 2.2.4 Characterisation of model particles

##### 2.2.4.1 Sample preparation

To examine the particles, the tablet shell was dissolved in water overnight on a platform shaker (Universal shaker SM A1, Edmund Bühler GmbH, Germany). The released particles were washed several times with water over a sieve with a mesh size of 710 μm and dried at 35 °C overnight.

##### 2.2.4.2 Optical appearance of model particles

The printed particles of six tablets were visually analysed using a digital microscope (VHX-7000, Keyence, Japan). The diameter of the lower end of the particle with respect to the printing order and the height of single particles were determined with the microscope (ten particles for each tablet).

##### 2.2.4.3 Determination of the presence of individual model particles

To determine whether individual particles were present after the dissolution of the tablet shell, the particles of 14 tablets from different batches were analysed regarding their individuality, using dynamic image analysis (CPA 2–1, Haver & Boecker OHG, Germany). All particles obtained from one tablet were measured once. In addition to the generated pictures of the particles the equivalent particle diameter was used to confirm the optical observation. A distinct increase in the equivalent particle diameter indicated the presence of agglomerates.

##### 2.2.4.4 Mass variation of single model particles

Ten single particles of six tablets (60 in total) were analysed for mass variability with an analytical balance (Cubis® II Semi-Micro Balance, Sartorius AG, Germany).

#### 2.2.5 Manufacturing and characterisation of pharmaceutical model API-loaded filament

The powder mixture for the hot-melt extrusion of model API-loaded filament for the MUPS particles was mixed in a blender (Turbula® T2F, Willy A. Bachofen Maschinenfabrik AG, Switzerland) for 15 min. A co-rotating twin screw hot-melt extruder (ZE 9 L/D 20:1, Three-Tec GmbH, Switzerland) with a 3.0 mm die was used to produce pharmaceutical grade filaments. The screw configuration consisted only of conveying elements. A gravimetric feeder (ZD 5 FB, Three-Tec GmbH, Switzerland) was applied for the powder feeding. A conveyor belt (Model 846,102.001, Brabender® GmbH & Co. KG Germany) was employed to continuously adjust the filament diameter as HPMC is known for a die-swelling behaviour (Colon et al., 2023; Tabriz et al., 2021). A subsequent laser module (Laser 2025 T, Sikora AG, Germany) was used to determine the filament diameter. Only filaments with a diameter of 2.85 ± 0.05 mm were used for 3D printing of tablets. The filaments were visually analysed using a digital microscope (VHX-7000, Keyence, Japan). The content of the powder mixture and the hot-melt extrusion parameters are listed in Table 2.

**Table 2:** Content of powder mixture for hot-melt extrusion and extrusion parameters for the API-loaded pharmaceutical filament.

**Content powder mixture (% w/w)**	
Hydroxypropyl methylcellulose (AFFINISOL™ HPMC HME 15LV)	79
Fumed silica	1
Paracetamol	20


**Hot-melt extrusion parameters**	

Temperature zone 1	120 °C
Temperature zone 2	165 °C
Temperature zone 3	165 °C
Extruder screw speed	30 rpm
Powder feed rate	80 g/h

#### 2.2.6 3D printing and characterisation of model API-loaded MUPS tablets

##### 2.2.6.1 3D printing of API-loaded MUPS tablets

To test the transferability of the proposed modular system, the model API-loaded HPMC filament was used for 3D printing of particles. The second filament for the tablet shell was the commercially available PVA filament previously used for the model 3D-MUPS. The overall printing parameters can be found in section 2.2.2. The parameters for the second print head were not changed, as the same commercial PVA filament was used for the tablet shell. Only the printing parameters for the first print head used for the particle printing had to be adjusted. A higher temperature of 205 °C was needed for the HPMC filament. The active levelling was deactivated, and the build plate was manually levelled before printing. For better material flow by both print heads after switching from PVA to HPMC and vice versa, a support structure in the form of a prime tower was printed. The HPMC filament used for particle printing was attached by filament welding to commercially available PLA filament. Filament welding, also called fusion bonding, is a method used to join thermoplastic materials (Yousefpour et al., 2016). Within the 3D printing area, for example, it is used to minimize the amount of filament waste by combining a nearly empty filament spool with a new one (Mitchell, 2023). In this case, the PLA filament was only used as a push filament to guarantee filament feeding and retraction of the HPMC filament but was not used for printing. The bonded HPMC/PLA filament was loaded into the 3D printer. Three tablets were simultaneously printed with API-loaded HPMC and PVA filament.

##### 2.2.6.2 Optical appearance of API-loaded MUPS tablets

The printed tablets were visually analysed using a digital microscope (VHX-7000, Keyence, Japan).

##### 2.2.6.3 In vitro dissolution testing of API-loaded MUPS tablets according to Ph. Eur. 2.9.3

Drug release studies of the API-loaded 3D-MUPS tablets were carried out according to monograph 2.9.3 and 5.17.1 of the Ph. Eur. (version: 11.1) using a basket apparatus (900 ml phosphate buffer pH 6.8, 37 °C, 100 rpm, SOTAX AT 7 smart, SOTAX AG, Switzerland). Samples (5 ml) were taken with media replacement at specific time points. The release studies were terminated at the plateau phase. Samples were filtered using 10 μm filters and properly diluted prior to analysis. Drug release was measured with a UV–VIS spectrometry microplate reader (Tecan Spark, Tecan Group AG, Switzerland) at a wavelength of 243 nm. The calibration curve ranged from 1.0 to 21.0 μg/ml (R^2^ = 0.9996). The amount of cumulative drug release (in mg) as a function of time was determined. Dissolution tests were carried out as triplicates.

## 3 Results and discussion

### 3.1 Computer model design of the MUPS tablets

Conventional MUPS preparations often consist of pellets that are compressed with excipients to form a tablet. The tablet shell serves to hold the individual particles together as a complete unit to simplify dosing and application. The conventional composition of MUPS tablets, which involves particles enclosed within a tablet shell, should also be utilised to produce 3D printed MUPS (3D-MUPS).

**Fig. 1:**
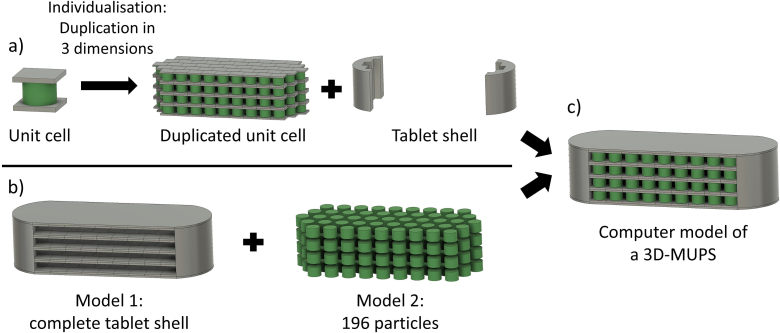
Computer models of the 3D-MUPS tablet, a) the modular system of the unit cell that was duplicated in all three dimensions and rounded off with an outer tablet shell, b) two separate computer models (tablet shell and particles) were generated for the dual extrusion printing process, c) the final computer model of a 3D-MUPS with 196 cylindrical particles with a size of 1.4 mm in diameter and 1 mm in height, size of the final tablet: 22.6 × 8.5 × 6 mm, green colour: particles, grey colour: tablet shell. (For interpretation of the references to colour in this figure legend, the reader is referred to the web version of this article.)

To realise 3D-MUPS tablets, two computer models had to be generated with CAD software: a model for the tablet shell and a model for the internal particles (Fig. 1a-b). One of the primary goals when modelling the tablet was to achieve the highest possible particle load per tablet to have a large range for dosing the API for future investigations. The tablet model was based on a modular system. A unit cell with a cylindrical body and rectangular upper and lower separation layers were designed (Fig. 1a). This unit cell enabled individual duplication in each of the three spatial directions to ensure simple dose adjustment in the case of personalised medicine by increasing the total amount of particles. The model presented differs from existing systems for oral dose adjustment of tablets. Korte et al. implemented the dose adjustment by changing the infill density of printed tablets (Korte and Quodbach, 2018). El Aita et al. adjusted the dose for paediatric use depending on the number of printed layers of cylindrical tablets, resulting in different tablet heights (El Aita et al., 2020). The cylindrical particle body of the proposed 3D-MUPS had a diameter of 1.4 mm and a height of 1 mm. The upper and lower rectangular separation layers for the particles had the dimensions of 1.7 × 1.8 mm. The separation layer was printed four times (layer height of 0.1 mm, Table 1) on top of the particles over the entire surface to ensure that the particles were 0.4 mm apart in the vertical direction (Fig. 1a). The size of the particles was chosen so that they were smaller than 2 mm to theoretically allow for a quicker stomach passage (Abdul et al., 2010).

The final model consisted of four layers in the z-direction. Each layer contained 49 cylindrical particles, resulting in a total number of 196 particles per tablet. The total volume of all particles in one tablet was 302 mm^3^. The final tablet model is only exemplary and was chosen with maximum particle loading. In the future, tablets with fewer layers or particles per layer can be modelled as required. The proposed model is based on uncoated matrix particles incorporated in a tablet-shaped body. However, compared to conventional MUPS tablets, these are not pharmaceutical pellets by definition, which are spherical, free-flowing objects with a narrow particle size and a diameter of 0.5 to 1.5 mm (Ghebre-Selassie, 1989). The particles' cylindrical shape and the particles' layered appearance caused by the 3D printing process contradict this definition. For this reason, this model is referred to as (matrix) particles and not as pellets.

To avoid sharp edges and increase swallowability, the tablet was rounded off with an outer tablet shell. This tablet shell consisted of curved walls on both endings and an upper and lower layer throughout the whole upper and lower surface. The final flat-faced oblong model used for all the experiments had the dimensions of 22.6 × 8.5 × 6 mm (Fig. 1c). The maximum size in the three spatial directions was determined based on medications available on the market. Marketed tablets for peroral use containing antibiotics or antivirals are often quite large tablets due to the high drug loadings, and the design of the 3D-MUPS was based on these. The tablet Prezista® 800 mg film-coated tablet by Janssen-Cilag GmbH for the treatment of human immunodeficiency virus (HIV), for example, has a tablet size of 20.0 × 10.2 × 7.6 mm (*ABDATA Plus X - Prezista 800 mg Filmtablette - Janssen-Cilag GmbH*, 2023). Hummler et al. investigated the swallowability of different shaped and sized tablets in adults. Among other things, they examined oblong tablets with a size of 20.5 × 8.89 × 6.07 mm (Hummler et al., 2023). Arafat et al. manufactured tablets with a maximum size of 19.5 × 6.6 × 7.1 mm (Arafat et al., 2018). However, the 3D-MUPS tablets will likely be difficult to swallow due to their size. As already shown by Bogdahn et al., the swallowability of tablets is also strongly dependent on the external shape, and geometries with corners and edges proved to be particularly difficult to swallow (Bogdahn et al., 2021). The modular system of the tablets allows the dose to be customised. Theoretically, tablets with a lower dose can be produced, which would also reduce the size of the tablet. The two generated computer models were separately loaded into the slicing software and precisely merged (Fig. 1c).

In order to ensure the intended gastric emptying behaviour, it is considered necessary that the particle size is below 2 mm, as this might be a possible threshold reported in literature (Newton, 2010). The distance between a particle and neighbouring particles in the x-y plane was 0.4 and 0.52 mm. A smaller distance led to particle fusion during the printing process. The tablet was intentionally designed to be open on the other sides to maximise the contact surface for subsequent tablet disintegration.

Awad et al. have already printed so-called miniprintlets with a 1- or 2-mm diameter using selective laser sintering (SLS) for multiparticulate systems. They were able to print 100 individual oral drug-loaded dosage forms (Awad et al., 2019). These miniprintlets would subsequently have to be processed into tablets/capsules. The model we propose combines these two steps in a single process by directly printing a complete tablet.

**Fig. 2:**
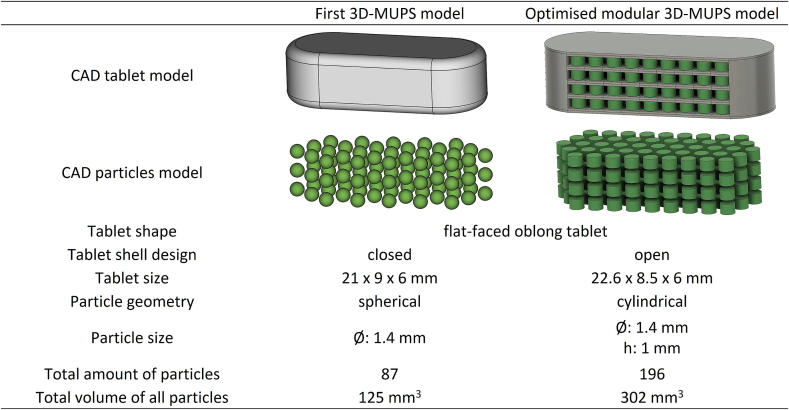
Comparison of the two different CAD models for 3D-MUPS.

The modular system 3D-MUPS described above was not the first model we evaluated in order to print 3D-MUPS. The first model had a completely filled tablet shell with cavities for the particles (Fig. 2). As a result, the proportion of shell material with respect to the entire dosage form was higher, which was considered counterproductive regarding the subsequent tablet disintegration. In addition, the tablet shell of the first model had a lower surface-area-to-volume ratio. Accordingly, individual particles were modelled for the optimised model that only came into contact with the shell material at the upper and lower surface layers. Preliminary tests with spherical particles with a diameter of 1.4 mm were also conducted. This particle shape was based on conventional MUPS preparations, which often contain spherical pellets. However, these investigations provided inconsistent results. On the one hand, the first and last layers could not be sliced precisely by the software and, therefore, could not be printed accurately. This reduced the quality of the final product. Secondly, switching to cylindrical particles increased the contact surface for subsequent tablet shell layers. In our experiments, this positively affected the adhesion of the tablet shell layers to the particles, as detected visually, to print a coherent, uniform dosage form. It is not expected that the cylindrical shape will impact gastric emptying. Data on emptying of small cylindrical particles is not available to the authors' knowledge. However, we assume that gastric emptying is not dependent on the shape but rather on the size and mechanical properties of the objects. However, this will have to be verified in future in vivo experiments. Further differences between the first computer model and the optimisation towards the current computer model are shown in Fig. 2. With the modular setup, the number of particles and the total volume of all particles were markedly increased while only slight changes in the outer dimensions of the tablet were necessary.

### 3.2 3D printing of model MUPS tablets

Compared to the conventional production of MUPS using a tablet press, FFF printing has the advantage that no compaction process takes place. This means less force is exerted on the particles, which could destroy them (Abdul et al., 2010). In addition, the pellets and the excipient mixture cannot segregate during the manufacturing process, as the particles are assigned a defined position in the computer model by printing them at a designated location in the tablet shell. One disadvantage, however, is the use of high temperatures during production. First, during the production of the filaments by hot-melt extrusion and again during the melting of the filaments in the print head. Therefore, only thermostable drugs or other excipients can be considered for this production method. There is no doubt that the throughput of tablet presses is a significant advantage over 3D printing, as well. With 3D printing, however, the focus is on patient-specific production. Personalised medicines, for example for geriatric patients, could improve therapy. Dose adjustments are often necessary to compensate for fluctuations in pharmacokinetics and different drug combinations are necessary for effective treatment of various diseases. MUPS can also be administered without a capsule or surrounding tablet shell after disintegration for geriatric patients with swallowing difficulties (Breitkreutz and Boos, 2007). Using multiple print heads also enables processing different polymer bases and/or APIs.

**Fig. 3:**
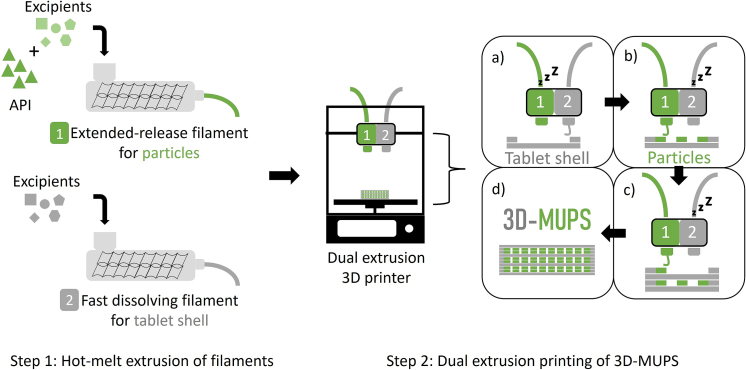
Manufacturing process for 3D printed MUPS (3D-MUPS) tablets, step 1: manufacturing of two different pharmaceutical filaments (without/with active pharmaceutical ingredient (API)) by hot-melt extrusion for the tablet shell and the particles, step 2: dual extrusion printing process for 3D-MUPS: a) print head 2 prints the tablet shell while print head 1 is inactive (symbol: Zzz), b) print head 1 prints the particles, c) layer by layer, the printer changes print heads to print either shell or particles alternately, d) The final product is a 3D printed MUPS tablet, green colour: particles, grey colour: tablet shell. (For interpretation of the references to colour in this figure legend, the reader is referred to the web version of this article.)

The developed manufacturing process for 3D-MUPS tablets consisted of two steps: the production of the pharmaceutical filaments by hot-melt extrusion and the dual extrusion 3D printing (Fig. 3). A dual extrusion 3D printer was used for the implementation. The two print heads were required to print filaments with different physicochemical properties simultaneously. One print head was used to print the tablet shell. A water-soluble polymer was selected to ensure a fast disintegration in the stomach and release of the embedded particles. The other print head was used to print the matrix particles. The particles can contain the active ingredient(s) and should consist of a polymer with sustained release properties. During the printing process, the two print heads were activated alternately, one after the other. This allowed the model to be generated layer by layer with different parts.

The proof-of-concept experiments focussed on printing the model 3D-MUPS and evaluating the developed computer model design. Therefore, commercially available filaments with highly standardised properties were used. PVA was used for the tablet shell material, and PLA was used for the particles. Print head 1 was loaded with PLA filament, while the other was loaded with PVA filament. The printing process started by printing the first layers of the tablet shell with print head two while the first print head was inactivated (Fig. 3 inlay a). After the first layers of the tablet shell were printed, the first print head was activated to print the first layer of particles on top and between the tablet shell (Fig. 3 inlay b). By activating the print heads and printing tablet shells or particles alternately, it was possible to print the model, which comprised 60 layers (Fig. 3 inlay c). The final product was a 3D printed MUPS tablet (Fig. 3 inlay d, 3D-MUPS).

Several printing parameters were identified to be critical to print a 3D-MUPS tablet. These parameters were optimised empirically and are described in Table 1. As 49 particles had to be printed on a small area with small gaps between the particles within one print layer, no polymer was permitted to leak from the print heads (oozing). Oozing out of the print head would have led to stringing between the particles, which would have led to particle agglomerates bigger than 2 mm. To prevent this, the settings were adjusted to retract the filament from the hot print head before moving from one particle to the next. This was achieved with the retraction option in the slicing software. The maximum retraction count per layer was set to a level of 300, and the minimum travel distance before a retraction was performed was set to 0 mm. As many retractions were performed throughout the printing process, slight filament abrasion by the gears was observed. As already reported in the literature, common pharmaceutical grade polymers that are interesting for 3D printing often lack the needed mechanical properties and are not printable (Nasereddin et al., 2018; Samaro et al., 2020; Solanki et al., 2018). The observed filament abrasion indicates that much force was being transmitted onto the filament, which may lead to breaking when switching to pharmaceutical grade filaments. The retraction of the filaments was also beneficial for the print heads while they were inactive. It was observed that the polymer tended to ooze from the nozzle during this time. These polymer residues adhered to the outside of the tablet when the print head was reactivated. This oozing from the nozzle in inactive mode could be reduced by activating the retraction.

**Fig. 4:**
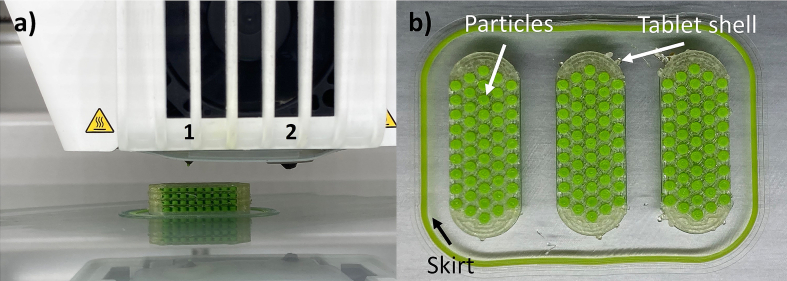
Pictures of the printing process of 3D-MUPS tablets, a) finished 3D-MUPS on the print bed of the dual extrusion printer UltiMaker S3, 1: print head with 0.25 mm nozzle for particles, 2: print head with 0.4 mm nozzle for tablet shell, b) in-process picture from the top while the printer changes from one print head to the other; tablet shell, single model particles and skirt support structures are visible.

For the precise printing of the small particles, a nozzle with a diameter of 0.25 mm was chosen. For the printing of the tablet shell, a bigger nozzle with a diameter of 0.4 mm was acceptable (Fig. 4a).

To improve the filament feeding, an auxiliary structure in the form of a skirt was printed at the start of the printing process (Fig. 4b). To prevent a uniform print seam from forming in the tablet shell, the print starting point for each layer was set to random. Commercially available water-soluble glue was used to improve the print bed adhesion of the first layers. This type of print bed adhesion was acceptable for the proof-of-concept study. For later production, however, the print bed adhesion would have to be assured by other methods.

On average, it took around one hour to print one tablet. This was due to the low printing speed and the high number of retractions, which were crucial for successful printing. By further optimising the printing parameters in future experiments, a reduction in printing time might be possible.

**Fig. 5:**
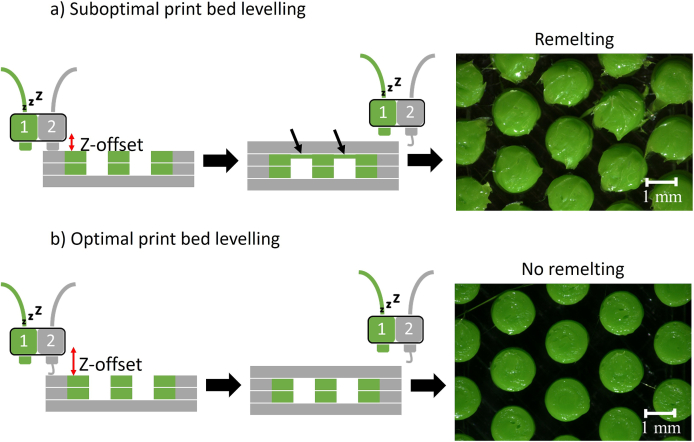
Importance of accurate print bed levelling for 3D printing of MUPS tablets, a) when the distance between the second print head and the print bed was too small (z-offset, red arrow) the second print head touched the already solidified particles, because of the high temperature of the print head, this led to remelting of the PLA, which resulted in a connection between the particles (black arrows), and particle agglomeration was obtained, b) in the case of the exact distance single particles are obtained, no remelting of the particles by the second print head is visible, green colour: particles, grey colour: tablet shell. (For interpretation of the references to colour in this figure legend, the reader is referred to the web version of this article.)

Another crucial parameter is the calibration of the distance of both print heads to the print bed (z-offset), also referred to as print bed levelling. After dissolving the tablet shell, it was found that, in some cases, the particles were not individual particles as planned but particle agglomerates. Thin films were visible between the individual particles, which led to a connection (Fig. 10c-d). According to the European Pharmacopoeia definition for optical particle characterisation, agglomerates are particles that are fused or cemented (Monograph 2.9.37: Optical microscopy, 2023). This definition can also be applied to 3D-printed particles, which is why this term is used throughout the text. A separate experiment was conducted to determine whether this was due to the remelting of the PLA particle surfaces by the PVA printhead while travelling across them. Three tablets per batch were printed, with only one of the three tablets receiving PVA top layers. It was found that only the tablets in which additional PVA layers were printed on the particles showed visible remelting of the uppermost PLA layers (Fig. 5). It was assumed that minor deviations from the optimal z-offset led to undesired print results: When the distance between the second print head and the print bed was too small, the chance of particle agglomeration increased. The second print head collided with previously solidified particles. The PLA was remelted upon contact due to the second print head's elevated temperature of 224 °C. The melted material was then dragged to the next particle, resulting in a thin polymer connection between particles and particle agglomeration in the final product (Fig. 5a, small black arrows). Furthermore, a combinatorial effect of the hot molten PVA and the hot print head could have led to a remelting and dragging of the PLA material. Individual particles only were obtained with proper print bed levelling (Fig. 5b, z-offset: red arrow).

**Fig. 6:**
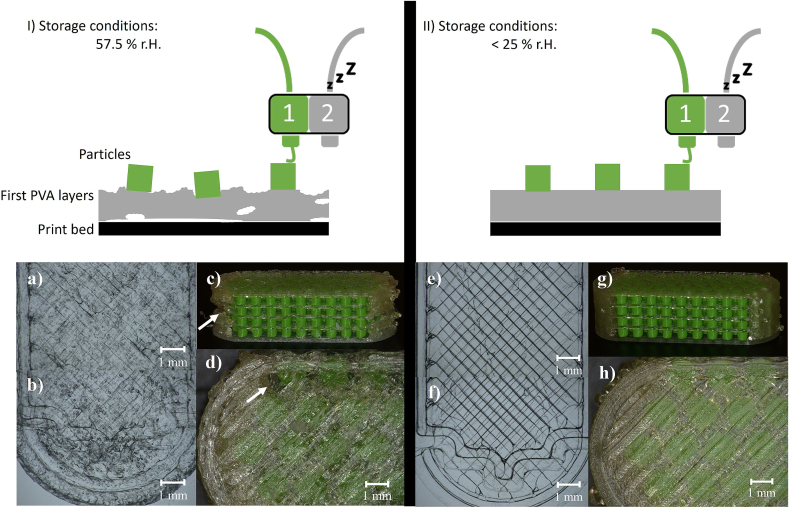
Effect of the storing conditions of the PVA filament on the print quality, I) storage conditions of 57.5 % relative humidity (r.H.) led to evaporation of entrapped water in the hot print head leading to imprecise printing, a-b) inhomogeneous first layers with visible entrapped air bubbles, c) 3D-MUPS with visible gaps in the tablet shell, d) rough tablet surface with gaps and air bubbles. II) storage conditions below 25 % r.H. inside filament storage box led to precise printing of PVA layers, e-f) homogenous first PVA layers, g) 3D-MUPS with good print quality, h) smooth tablet surface, pictures a-b and e-f: images were taken with light from underneath and above the object to make the layers translucent, white arrows: gaps in tablet shell.

When printing tablets with two nozzles, Kempin et al. observed remelting of the material already deposited to the print bed by the second print head. The melted material was dragged along with the print head, which led to a deformation of the tablet (Kempin et al., 2018). As the fine adjustment of the print bed can only be guaranteed to a limited extent by the active bed levelling and cannot be completely ruled out, the chance of agglomerated particles is always given. A possible strategy against this problem might be using a polymer for the tablet shell that melts at lower temperatures than the particles. In that case, the temperature of the second print head should not be high enough to remelt the particles. However, a possible drawback of this approach could instead be the remelting of the PVA base plate by the first print head while the first particle layer is being printed. This strategy will be investigated in future experiments. Furthermore, the authors observed that the storage conditions for the PVA filaments influenced the quality of the obtained printed objects. Undesirable printing results were observed, particularly in the summer when a higher relative humidity was measured. To analyse the effect of the storage condition on the print result, the filaments were stored at <25 % r.H. or 57.5 % r.H. The PVA filament stored at higher relative humidity resulted in uneven polymer strands upon printing, most likely caused by bubbles forming inside the print head when heated to 224 °C (Fig. 6). This is likely due to water evaporating from the filament. As a result, the printed PVA layers on the print bed were inconsistent and inaccurate (Fig. 6a-b). This was especially problematic for the first layers, as the particles could not be printed at a straight level when the PVA layers were uneven (Fig. 6 I). This misalignment of the particles then affected all subsequent print layers, leading to undesirable print results for the tablet. Defects in the tablet shell became visible, and the tablets had a rough surface with visible gaps (Fig. 6c-d). Further, the printing process had to be terminated several times because no filament was being extruded. Printing with PVA filament stored below 25 % r.H. in a filament box equipped with silica gel, however, led to more reliable results (Fig. 6 II): The first PVA layers were homogenous with no entrapped bubbles (Fig. 6e-f) and appeared translucent in the microscopic images. Printing tablets with continuous PVA layers without gaps and with overall good print quality without printing interruptions was possible. Only small PVA residues were visible on the outside (Fig. 6g-h).

Laumann et al. investigated how different degrees of humidity affect different hygroscopic polymer filaments. They found that moisture influences the adhesion of the printed objects to the print bed. This was observed in particular for the hygroscopic PVA (Laumann et al., 2023). Even though the adhesion of the PVA tablet shell was good, this shows that the filaments' water content can influence the printing process. Accordingly, all filaments were stored in a sealed filament box, in which the relative humidity was kept below 25 % by adding silica gel as a desiccant. The filament was fed through a tube directly from the box into the print head to avoid contact with the surrounding environment.

### 3.3 Characterisation of model MUPS tablets

**Fig. 7:**
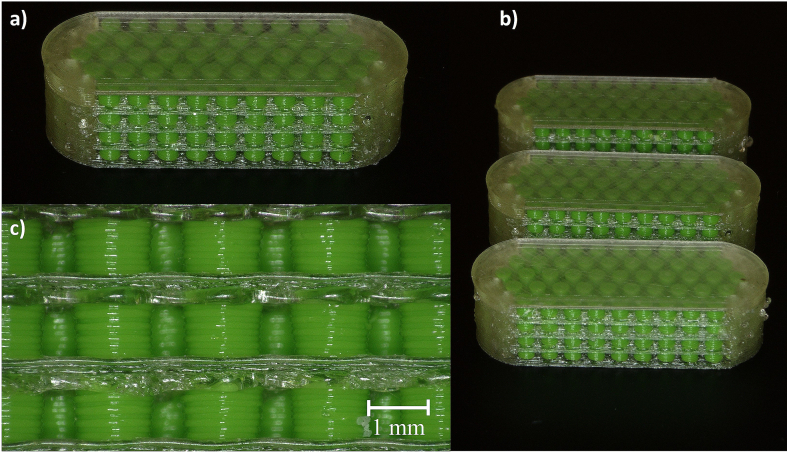
Microscopic pictures of 3D-MUPS model tablets, a-b): the incorporated particles were visible through the gaps and the translucent PVA tablet shell, small PVA residues are visible, pictures taken at an angle of 60°, c): the shape of particles and intermediate PVA separation layers, pictures taken at an angle of 90°.

The printed tablets had a uniform print quality. Only small PVA strings were visible on the outside of the tablets, which resulted from oozing out of the inactive print head (Fig. 7a-b). They were loosely attached to the tablet shell and could be easily removed. The single particles were visible through the gaps and translucent PVA shell and were of the same shape. The separation layers of PVA in between the particles showed small irregularities and were not as straight and regular as the following layers (Fig. 7c). As shown in Fig. 4, the PVA separation layers on top of the particle layers had to close the gaps between the particles. Since the melted PVA can also migrate into the gaps, this may have resulted in an uneven first separation layer. This irregularity was probably corrected with subsequent layers. The tablets had a smooth and homogenous top and bottom side.

**Fig. 8:**
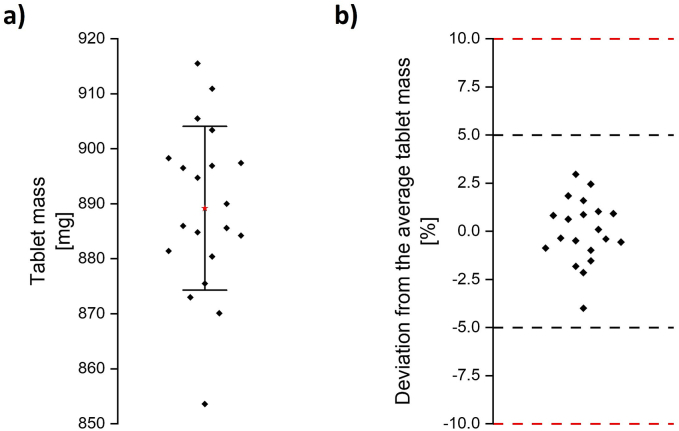
Characterisation of the tablets regarding the tablet mass, a) tablet mass of printed tablets, *n* = 20, red star: x, error bar: standard deviation, b) uniformity of mass of single-dose preparations according to European Pharmacopoeia 2.9.5 with deviations from the average tablet mass, red and black dashed lines: 5 and 10 % limits according to the European Pharmacopoeia. (For interpretation of the references to colour in this figure legend, the reader is referred to the web version of this article.)

The tablets had an average mass of 889.2 ± 14.9 mg (Fig. 8a, red star). According to the European Pharmacopoeia 2.9.5 “Uniformity of mass of single-dose preparations”, for tablets weighing more than 250 mg, only a maximum of two tablet masses may deviate by more than 5 %, and no tablet mass may deviate by more than 10 % from the average mass. No tablet mass deviated more than 5 %. The highest deviation from the average tablet mass was observed for one tablet with - 4 % (Fig. 8b). The 3D-MUPS tablets, therefore, met the requirements of the European Pharmacopoeia. In future, tablets containing an API must comply with the monograph on content uniformity of the European Pharmacopoeia (2.9.40, “Uniformity of dosage units”).

**Fig. 9:**
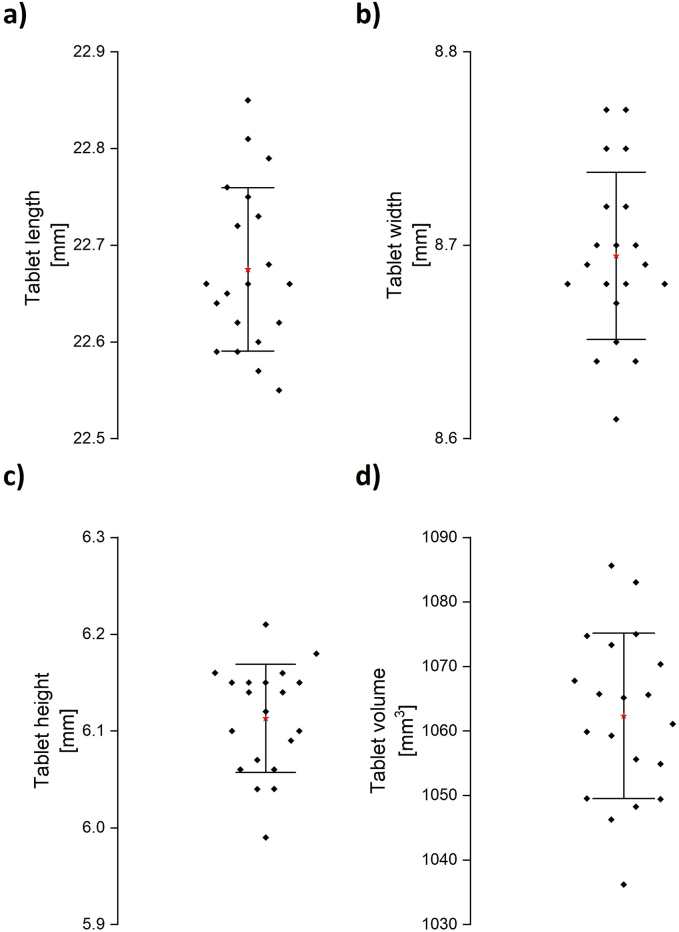
Tablet length, width, height and volume of 3D-MUPS tablets, n = 20, red stars: x, error bars: standard deviation, size of the computer model: 22.6 × 8.5 × 6 mm, volume of computer model: 1019.2 mm^*3*^. (For interpretation of the references to colour in this figure legend, the reader is referred to the web version of this article.)

The tablets had the desired shape and size of the computer model (22.6 × 8.5 × 6 mm), and only slight deviations were visible (Fig. 9). The tablets had an average length of 22.68 ± 0.08 mm, an average width of 8.69 ± 0.04 mm and an average height of 6.11 ± 0.06 mm (means ± standard deviation). The small deviations in size and mass showed that the printing process was sufficient for the reproducible manufacturing of 3D-MUPS tablets. For better comparability, the volume of the tablets was calculated using the determined dimensions. The nominal volume of the computer model was 1019.2 mm^3^. The printed tablets had an average volume of 1062.3 mm^3^ (Fig. 9d). Therefore, tablets had an average volume of around 4.2 % more than the computer model. The small deviations in the tablet size and the resulting larger calculated tablet volume could have been caused for example by over-extrusion during the printing process and by smallest deviations from the ideal filament diameter.

Crushing resistance and friability tests were carried out to analyse the mechanical properties of the 3D printed tablets. None of the ten tablets tested were found to break in the resistance to crushing experiment. In the friability experiments, no dust due to abrasion was visible and no mass deviation was detectable. However, the base plates of two tablets detached during the friability testing and one particle separated from the delivery system in each case. This can be explained by the fact that the tablets have to be removed from the print bed at the end of the printing process. As it is not possible to simply detach the tablets, a lot of mechanical energy has to be applied. The base plate of the tablet sometimes partly detaches from the rest of the tablet. However, the integrity of the tablet remains intact and no particles detach during removal. This defect most likely caused the base plates to detach while testing for friability. 8 out of 10 tablets remained completely intact. The experiments indicate good mechanical stability of the 3D-MUPS tablets.

### 3.4 Characterisation of model particles

**Fig. 10:**
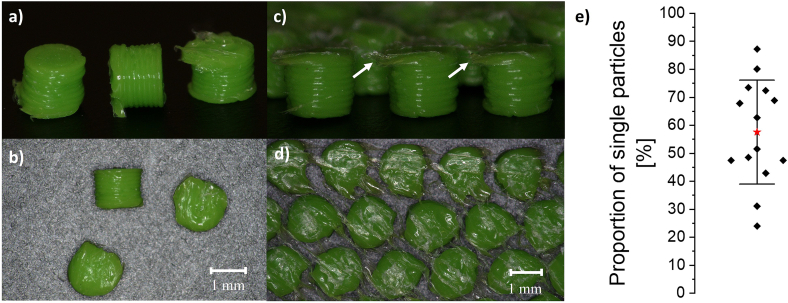
Characterisation of the particles with regard to the presence of single particles or agglomerates after dissolving the tablet shell in water, a-b) microscopic pictures of single model particles at a 70° angle and from above, c-d) microscopic pictures of agglomerated model particles at a 70° angle and from above, connections between the particles were visible in the uppermost layers (arrows), e) proportion of single particles within one tablet of 14 selected tablets by dynamic image analysis, red star: x, error bar: standard deviation. (For interpretation of the references to colour in this figure legend, the reader is referred to the web version of this article.)

To determine whether it was possible to set free single particles from the model 3D-MUPS, the tablet shell was completely dissolved in water. Both single (Fig. 10a-b) and connected/agglomerated particles (Fig. 10c-d) were visible under the microscope. It is noticeable that the connections that form between the particles only occur in the uppermost layer of the PLA particles. In previous tests with the first tablet model (Fig. 2), in which the tablet shell was also printed between the particles instead of just a bottom and top layer, connections were visible over the entire height of the particles (data not shown). This indicates that the connections between the particles were most probably due to remelting of the uppermost PLA layers when the hot PVA nozzle printed the subsequent layer on top of the particles, as previously discussed (section 3.2 and Fig. 5). Traces of PLA remelting were also visible on the individual particles (Fig. 10a). However, these particles have not formed a continuous connection to the next particle, or this connection may have been removed in subsequent processing steps (e.g. dissolving the tablet shell). The connections between the particles were very loose and broke easily. It might be possible that because of the stomach's motility, those loosely connected particles might be separated again.

Dynamic image analysis was used to quantify the number of single particles and particle agglomerates. The generated images were checked visually for individual particles. In addition, the equivalent particle diameter was used to support the optical observation. In the analysis of 14 selected tablets of different print batches, an average of 57.54 ± 18.59 % of single particles was measured. The lowest percentage measured was around 24 %, and the highest was 87 % (Fig. 10e). An explanation for the high variability might be the calibration of the print bed, which was done each time before printing a new batch, resulting in slightly different z-offset values. As already mentioned, minimal deviations in the z-offset lead to the PVA head remelting the PLA particles, causing the particles to agglomerate (Fig. 5). Since the calibration of the print bed cannot be checked using the printer's software, it is assumed that this is the reason for the deviations. Further research and optimisation of the printing model should reduce the occurrence of agglomerates.

**Fig. 11:**
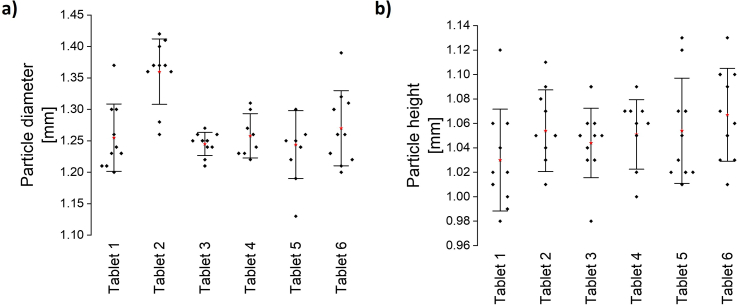
Characterisation of single model particles (*n* = 10) with regard to the particle shape after dissolution of the tablet shell, a) particle diameters, b) particle heights, all obtained from ten individual particles for six individual tablets, red stars: x, error bars: standard deviation, size of the computer model: 1.4 mm diameter, 1.0 mm height. (For interpretation of the references to colour in this figure legend, the reader is referred to the web version of this article.)

When examining tablets with primarily single particles, uniform particles were obtained. The particles had the desired cylindrical shape of the computer model, and as expected, ten single printing layers per particle were clearly visible (Fig. 10a). The particles of all six tested tablets had an average diameter of 1.27 ± 0.04 mm and an average height of 1.05 ± 0.01 mm (Fig. 11a-b). Therefore, the particles were slightly smaller in diameter but bigger in height compared to the computer model (1.4 mm diameter, 1.0 mm height).

**Fig. 12:**
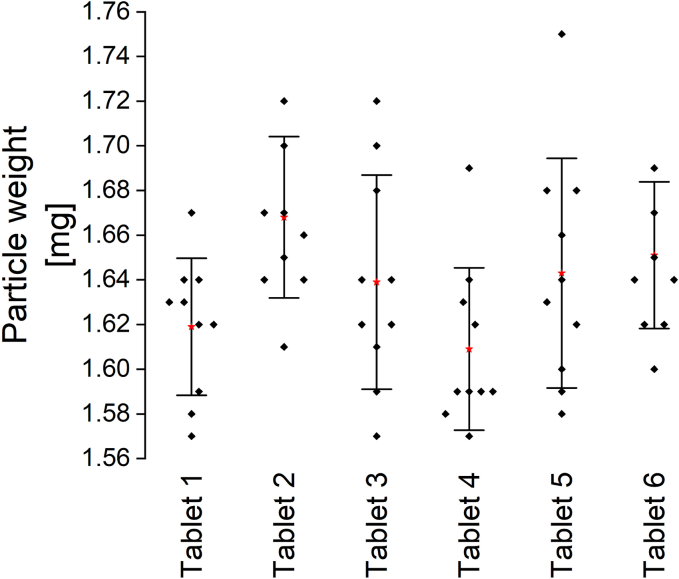
Particle weight of single model particles (n = 10) after the dissolution of the tablet shell, all obtained from ten individual particles for six individual tablets, red stars: x, error bars: standard deviation. (For interpretation of the references to colour in this figure legend, the reader is referred to the web version of this article.)

The average particle weight determined for ten particles from each of the six tested tablets was 1.64 ± 0.02 mg (Fig. 12).

Krause et al. printed cylindrical minitablets with 4.0, 3.0, 2.0- and 1.5-mm diameters. They concluded that the smaller the tablet diameter, the more inaccurate and imprecise the printing of the finished tablets became, which was due to the use of FFF printing in general. The 1.5 mm mini tablets had a diameter of 1.3 ± 0.1 mm and a height of 0.93 ± 0.07 mm (target height: 1.0 mm). The tablets had a mass of 2.47 ± 0.34 mg. In comparison, it is noticeable that these tablets were also smaller than the computer model. Overall, the fluctuations of Krause et al. are more prominent, but this is probably because a different polymer was used here, which was also extruded in-house. The commercial filament used for the 3D-MUPS presumably has smaller fluctuations in diameter, which in turn has a positive effect on the printing result. In summary, it can be said that the results of Krause et al. are consistent with the present results. Interestingly, these tablets also showed thin polymer films on the top side, which optically resemble the connections between the agglomerated particles after the dissolution of the tablet shell (Krause et al., 2021). Awad et al. used selective laser sintering to print spherical particles (miniprintlets) with a 2.0- and 1.0-mm diameter. The results of the visual analysis of the diameter and the weight analysis are consistent with the results for 3D-MUPS. Although two different printing methods were used, uniform particles with comparable standard deviations were obtained. Our results are consistent with the observation that printing small particles using FFF is possible. However, printing particles smaller than 1.4 mm, for example, done by Awad et al., might be problematic using FFF. However, since a size of <2 mm is sufficiently small to shorten the time for gastric passage, it is questionable whether a further reduction in particle size is necessary for gastric emptying (Abdul et al., 2010). The influence of the particle size on drug release remains to be investigated in the future.

### 3.5 Manufacturing and characterisation of pharmaceutical model API-loaded filament

**Fig. 13:**
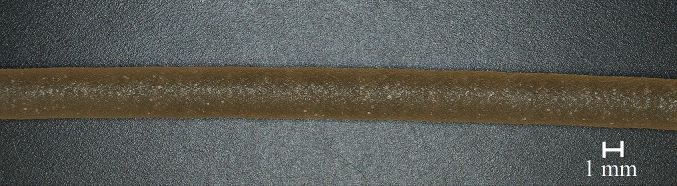
Microscopic picture of hot-melt extruded HPMC filament with 20 % of paracetamol, filament had a brown colour with a homogenous diameter and a rough surface. (For interpretation of the references to colour in this figure legend, the reader is referred to the web version of this article.)

3D printing of MUPS tablets with commercially available filaments showed good results regarding, for example, the printability of small particles. Therefore, a transferability study was conducted for the proposed modular system with pharmaceutical model API-loaded filament. HPMC was chosen as a polymer for the particles as it was already used several times successfully for hot-melt extrusion and 3D printing (Kadry et al., 2018; Krause et al., 2021; Melocchi et al., 2016; Prasad et al., 2019; Zhang et al., 2017). With the selected extrusion parameters (Table 2), it was possible to produce uniform model API-loaded filaments. The filaments had a brown colour and a rough surface (Fig. 13). PCM was used only as a model drug which has been used for FFF printing before and will have to be replaced with a more suitable API for the concept of MUPS tablets.

The diameter was monitored with a laser unit during the manufacturing process, and only filaments with a diameter of 2.85 ± 0.05 mm were used for further 3D printing of tablets. The highest extrusion temperature of 165 °C was below the reported degradation temperature of HPMC. Svoboda et al. analysed the thermogravimetric mass loss of AFFINISOL™ HPMC HME 15LV in air and concluded a three-stage mass loss with the highest loss of 70 % around 200 °C. They concluded a maximum extrusion temperature of 170 °C (Svoboda et al., 2023). The melting temperature of PCM is reported at 168–172 °C (Monograph Paracetamol, 2023). Therefore, small white particles were visible in the filament. So far, only the filament production for the particles was analysed. The production of the pharmaceutical grade filament for the tablet shell was omitted but will be established in the future with hydrophilic and fast-dissolving polymers such as polyvinylpyrrolidone or polyvinyl alcohol-polyethylene glycol graft-copolymer.

### 3.6 3D printing and characterisation of model API-loaded MUPS tablets

The HPMC filament was used for 3D printing of API-loaded MUPS tablets. The filament was flexible enough for printing through the Bowden tube setup. As discussed in chapter 3.2 filament retraction by the feeding gears proved to be an essential parameter during 3D printing of the model 3D-MUPS with commercial filaments. As the extruded HPMC filaments were too short to come into contact with the feeding gears, commercially available PLA filament was used to transfer the retractions by the gears onto the HPMC filament. This feeding concept of short filament pieces through a Bowden tube has already been used in literature (Domsta et al., 2023). For this concept to work, the PLA and HPMC filaments must be bonded together and function as a combined filament. Filament welding was used to attach the HPMC filament to the PLA filament. One advantage of this feeding concept is that abrasion of the HPMC filament is eliminated, as the force of the gears only acts on the PLA filament, which is not used as a printing material in this case.

**Fig. 14:**
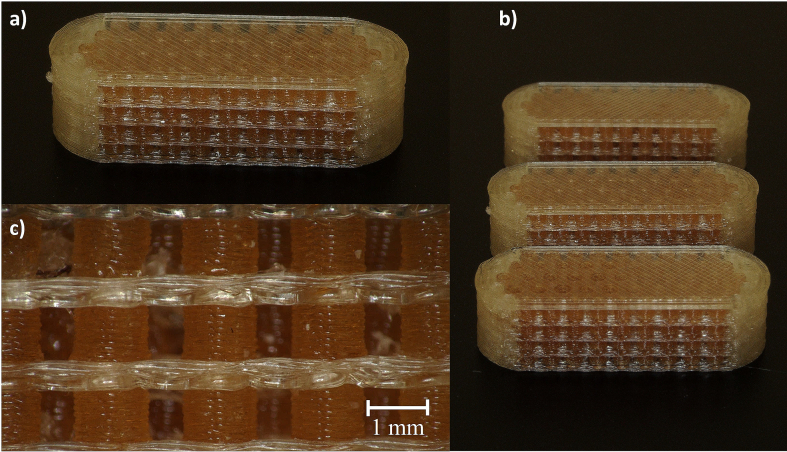
Microscopic pictures of 3D-MUPS tablets with paracetamol particles, a-b): the incorporated brown particles were visible through the gaps and the translucent PVA tablet shell, pictures taken at an angle of 60°, c): the shape of paracetamol particles and intermediate PVA layers, pictures taken at an angle of 90°. (For interpretation of the references to colour in this figure legend, the reader is referred to the web version of this article.)

3D printing of small particles with pharmaceutical filaments resulted in problems similar to those with commercial filaments. The right z-offset between the nozzles and print bed was again a crucial parameter. First printing experiments had to be aborted because of bad print results, especially the PVA layers that were not printable on top of the first layer of particles. This was probably due to the second nozzle being too close to the already printed particles. The already solidified particles might have blocked the PVA nozzle so that no PVA could be deposited on top of the particles. Adjustment of the print bed by manual levelling resulted in the successful printing of API-loaded 3D-MUPS with good printing quality (Fig. 14). So far, only commercially available PVA filament has been used for the tablet shell. Similar to the model MUPS tablets (Fig. 7c), the PVA layers between the particles were not as straight and regular as the following PVA layers (Fig. 14c). Nevertheless, the entire proposed modular system was printable with the API-loaded HPMC and commercially available PVA filaments (Fig. 14a-b).

The colour of the particles changed with regard to the colour of the extruded filament. The browner colour of the particles might be due to the second heating process with higher temperatures while 3D printing. Compared to the printed mini tablets of Krause et al., who worked with the same polymer and very similar extrusion and printing parameters, no noticeable colour difference was visible (Krause et al., 2021). Also, Prasad et al. obtained similar coloured brown HPMC filaments and tablets containing 5 to 35 % of PCM (Prasad et al., 2019). The three printed tablets had the same visual properties under the microscope (Fig. 14b), but further characterisation methods, as with the model MUPS tablets, must be established in the future.

**Fig. 15:**
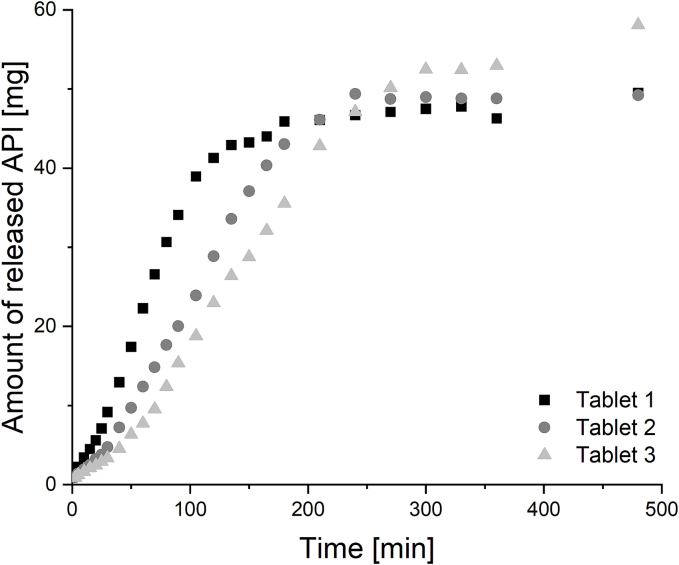
Individual dissolution profiles of 3D-MUPS tablets with PCM-loaded HPMC particles and PVA tablet shell (basket apparatus, 900 ml phosphate buffer pH 6.8, 37 °C, 100 rpm).

In vitro dissolution studies have shown that on average 80 % of PCM is released within 3 h, based on the maximum amount of API detected (Fig. 15). However, fluctuations were observed between the individual tablets (80 % API: 2–4 h). The maximum amount of PCM released was between 49 and 58 mg. During dissolution testing, it was observed that the tablets maintained their general tablet shape and were not present as individual particles but as a particle conglomerate. This phenomenon was probably caused by the slow dissolution of the PVA shell. The single particles were probably held together by sticky PVA residues. Initial studies on the disintegration time of the 3D-MUPS model tablets indicate that the tablets do not disintegrate within a reasonable time frame (data not shown). A fast disintegration time of MUPS tablet in general is essential to release the single particles it contains. Accordingly, this property must be adapted in the future for 3D-MUPS by using faster dissolving pharmaceutical grade filaments instead of commercially available PVA filaments for the shell. Initial release studies highlight the proof-of-concept approach with a model drug and that further optimisation is required to ensure that it can be used effectively for pharmaceutical applications. However, the first transferability studies suggest that 3D printing of API-loaded 3D-MUPS tablets with the proposed modular system is possible.

## 4 Conclusion

Compared to the conventional production of MUPS tablets with tablet presses, 3D printing seems to be advantageous as no compression forces are applied and easy dose adjustment is possible. We have therefore created a modular computer-aided design model of a 3D printable MUPS tablet with 1.4 mm diameter particles. To print this computer model, a dual extrusion 3D printing system was developed in which two nozzles print either the tablet shell or the incorporated particles. The setup was successfully tested as a proof-of-concept study using commercially available PVA and PLA filaments, and critical parameters were determined. The printed model tablets were characterised, revealing the problem of particle agglomeration. In a final step, the concept was successfully tested with a hot-melt extruded model API-loaded filament to check the transferability and suitability for the pharmaceutical sector. The first results showed that dual extrusion printing is a possible new manufacturing method for MUPS tablets. However, there are other limitations compared to the conventional method that make it a difficult and complex new manufacturing method to successfully print personalised MUPS tablets. These limitations and challenges include the momentarily long printing times and the avoidance of particle agglomerates. Further research will be conducted in which more suitable pharmaceutical grade filaments will be extruded for both the tablet shell (e.g. polyvinyl alcohol-polyethylene glycol graft-copolymer) and the particles (e.g. polymethyl methacrylate).

## CRediT authorship contribution statement

**Lee Roy Oldfield:** Writing – review & editing, Writing – original draft, Visualization, Project administration, Methodology, Investigation, Formal analysis, Data curation, Conceptualization. **Aaron Felix Christofer Mentrup:** Writing – review & editing, Conceptualization. **Stefan Klinken-Uth:** Writing – review & editing, Conceptualization. **Tobias Auel:** Writing – review & editing, Project administration, Methodology, Conceptualization. **Anne Seidlitz:** Writing – review & editing, Supervision, Project administration, Methodology, Data curation, Conceptualization.

## Declaration of competing interest

The authors declare that they have no known competing financial interests or personal relationships that could have appeared to influence the work reported in this paper.

## Data Availability

Data will be made available on request.
